# Genetic Diversity and Disease Resistance Genes Profiling in Cultivated *Coffea canephora* Genotypes via Molecular Markers

**DOI:** 10.3390/plants14172781

**Published:** 2025-09-05

**Authors:** Ana Carolina Andrade Silva, Letícia de Faria Silva, Rodrigo Barros Rocha, Alexsandro Lara Teixeira, Bruno Grespan Leichtweis, Moysés Nascimento, Eveline Teixeira Caixeta

**Affiliations:** 1 Institute of Biotechnology Applied to Agriculture (Bioagro), Federal University of Viçosa, Viçosa 36570-900, MG, Brazil; ana.c.andrade@ufv.br (A.C.A.S.); leticiafaria785@gmail.com (L.d.F.S.); bruno.leichtweis@ufv.br (B.G.L.); 2 Embrapa Café, Brazilian Agricultural Research Corporation, Brasília 70770-901, DF, Brazil; rodrigo.rocha@embrapa.br (R.B.R.); alexsandro.teixeira@embrapa.br (A.L.T.); 3 Institute of Research, Technical Assistance and Rural Extension of Espírito Santo (INCAPER), Vitória 29052-010, ES, Brazil; 4 Department of Statistics, Federal University of Viçosa, Viçosa 36570-900, MG, Brazil; moysesnascim@ufv.br

**Keywords:** SNP, genetic diversity, Conilon, Robusta, CBD, *Hemileia vastatrix*, genetic structure

## Abstract

Knowledge of the genetic diversity and resistance genes of *Coffea canephora* genotypes is essential to identify genetic resources that are better adapted to current climate conditions. This study aimed to molecularly characterize and evaluate the genetic diversity of coffee plants cultivated in Rondônia (Amazonia), Brazil, using SNP molecular markers, and to identify plants carrying resistance genes to two major coffee diseases: rust (*Hemileia vastatrix*) and coffee berry disease (CBD; *Colletotrichum kahawae*). Genetic diversity analysis revealed five main groups: Group II included 33 genotypes, primarily of the Robusta botanical variety; Group III contained 18 genotypes of the Conilon variety; Group V, the largest, comprised 85 genotypes, mostly hybrids between Robusta and Conilon. Groups I and IV showed fewer, more divergent genotypes. Molecular markers linked to resistance genes enabled the identification of clones with pyramided resistance alleles for both diseases. Three genotypes exhibited a complete pyramided configuration, while others showed different combinations of resistance loci. Marker patterns also allowed classification of genotypes based on origin, variety, and genealogy. These findings provide a valuable foundation for guiding crosses in breeding programs aiming to develop disease-resistant and climate-resilient clones and hybrids, while also supporting cultivar and clone traceability.

## 1. Introduction

Understanding the available coffee genetic resources is one of the pathways to developing new cultivars resilient to emerging climatic conditions, such as high temperatures and irregular rainfall [1,2,3]. Among the more than 124 *Coffea species* already described [4], *Coffea canephora* stands out for its wide geographical distribution in tropical regions worldwide [5]. It is a perennial, long-cycle, diploid plant (2n = 2x = 22), easily vegetatively propagated, and exhibits gametophytic self-incompatibility [6,7].

This species is characterized by two botanical varieties: Conilon and Robusta, both of which are cultivated and commercially traded [8]. The Conilon variety is characterized by smaller coffee plants that are drought-tolerant but more susceptible to diseases. In contrast, the Robusta variety comprises larger, more vigorous coffee plants with bigger leaves and fruits, exhibiting lower tolerance to water deficit but greater resistance to pests and diseases [9,10]. Genetic materials of the Conilon variety predominate in the Southeast Region of Brazil compared to Robusta, with the latter being more prominent in plantations in the North Region [11,12].

The term ‘botanical variety’ refers to individuals of the same species that have developed naturally and exhibit characteristics distinct from other individuals within that species [13]. Due to their divergent and complementary traits, the heterosis observed in crosses between Robusta and Conilon coffee plants can be utilized in breeding programs. This enables the development of hybrids that combine the best characteristics of both botanical varieties, such as greater drought tolerance (derived from Conilon) and enhanced resistance to pests and diseases (derived from Robusta) [14]. Therefore, the increased vigor seen in inter-varietal crosses can be effectively harnessed in cultivation [15,16].

Interest in the production and commercialization of *C. canephora* is steadily increasing worldwide. This species possesses organoleptic and chemical characteristics that are notably utilized in the production of instant coffee and in the development of blends with *C. arabica* [17,18]. *C. canephora* stands out in espresso blends, contributing to body and density [19,20]. Within the genus, it is considered one of the species best adapted to current climate changes, which are characterized by high temperatures, variability in rainfall, and an increased occurrence of pests and diseases [3,5,9].

In Brazil, the state of Rondônia stands out as the second largest producer of *C. canephora* [21]. Its coffee plantations consist of cultivars developed by the Embrapa Rondônia breeding program [10,16], as well as clones selected by local producers [6]. Understanding the genetic diversity and disease resistance genes present in these materials can guide Embrapa Rondônia and other breeding programs in the country in selecting and combining materials that better adapt to current climate changes.

Although intensively grown, these genetic materials are unknown in many aspects. Thus, the use of SNP molecular markers to estimate genetic diversity at the DNA level is a promising tool. These can be used to assess molecular genetic diversity, as they are the most abundant type of polymorphism in genomes and are codominant and biallelic [11,22].

Over the past decades, different classes of molecular markers have been employed to investigate genetic diversity in *C. canephora*, each contributing to the understanding of its complex population structure [23,24]. Dominant markers such as RAPD and AFLP were initially used for rapid diversity surveys, while codominant markers including SSR provided greater resolution for assessing allelic variation and population differentiation [25,26]. These approaches generated important insights into the genetic relationships among genotypes and supported the first steps in Robusta coffee breeding [27]. More recently, the development of high-throughput genotyping technologies has enabled the use of single nucleotide polymorphisms (SNPs), which offer higher density, genome-wide coverage, and reproducibility compared to earlier marker systems [28,29].

In this context, the characterization of genetic diversity is essential for understanding the origin and identity of the most cultivated genotypes. Genetic polymorphisms allow for determining both the similarity and identity of each evaluated genotype. Genetic similarity provides a foundation for better understanding the relationships between cultivated genetic materials, while individual identity enables the discrimination of one material from another, linking it to its field designation [30].

This genetic diversity can also be investigated using molecular markers associated with the main diseases affecting the crop [23,24,31,32]. Two significant diseases are coffee rust, caused by the fungus *Hemileia vastatrix*, and coffee berry disease (CBD), caused by the fungus *Colletotrichum kahawae* [33]. Although *C. canephora* carries resistance genes, studies indicate that there are varying levels of resistance among genotypes, including resistant, moderately resistant, and even susceptible coffee plants [34,35].

The objectives of this research were twofold: (i) to utilize SNP molecular markers to assess the genetic diversity of cultivated coffee plants from the Embrapa breeding program and those selected by coffee producers, and (ii) to apply molecular markers for identifying genotypes that possess various resistance genes against the primary diseases affecting the crop.

## 2. Results

### 2.1. Genetic Diversity

**Figure 1:**
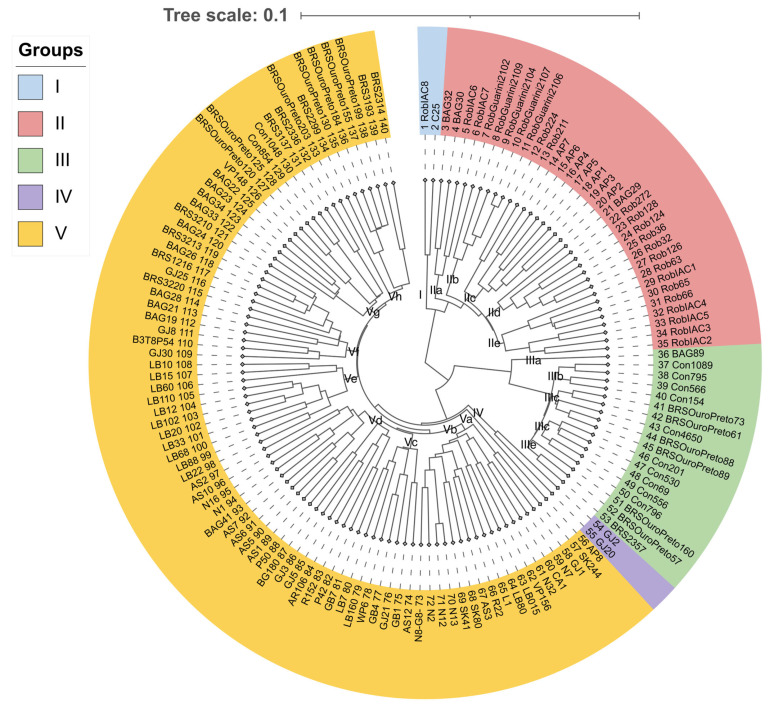
Dendrogram generated from the analysis of 39,329 SNP markers using hierarchical clustering with the Unweighted Pair Group Method with Arithmetic Mean (UPGMA), based on the dissimilarity matrix from the unweighted pairwise complement index of 140 genotypes of *Coffea canephora*.

In the quality analysis, SNPs with values below the thresholds were removed, resulting in a set of 39,329 markers distributed across the entire *C. canephora* genome. The polymorphic information content (PIC) of these markers ranged from values close to 0 up to the theoretical maximum of 0.375, with a mean of 0.220, indicating a moderate level of informativeness of the SNP panel. Based on these polymorphisms, a genetic dissimilarity matrix was generated and subsequently used to construct a dendrogram using the UPGMA algorithm (Figure 1). This clustering revealed the formation of five groups, three major and two smaller ones.

Group I consisted of two coffee plants (RobIAC8 and C25). Group II included 33 coffee plants and could be divided into five subgroups (IIa, IIb, IIc, IId, and IIe). Group III was made up of 18 coffee plants and, like Group II, could also be divided into five subgroups (IIIa, IIIb, IIIc, IIId, and IIIe). Group IV, similar to Group I, contained only two coffee plants (GJ2 and GJ20). The last and largest group, Group V, comprised 85 coffee plants and could be subdivided into eight subgroups (Va, Vb, Vc, Vd, Ve, Vf, Vg, and Vh).

In Group II, most coffee plants from the Robusta botanical variety were clustered. Of the 33 coffee plants identified with a Robusta passport, 30 were placed in this group. RobIAC8 was positioned in Group I, which is genetically the closest to Group II. Thus, the Robustas were grouped separately from the other varieties. The accession identified as Apoatã AP8 (individual 56) was grouped separately, indicating that this coffee plant may be a hybrid rather than a Robusta, as indicated by its passport. The Robustas in this group were introduced by the Agronomic Institute of Campinas (IAC) and are maintained in Embrapa Rondônia’s germplasm bank (BAG). This group includes accessions from IAC Collections 5, 10, 1655, 2258-1, and 2286, as well as Guarini and Apoatã IAC accessions. The Guarini coffee plants (RobGuarini 2102, RobGuarini 2109, RobGuarini 2104, RobGuarini 2107, and RobGuarini 2106) were grouped into subgroup IIb, while the seven Apoatã plants were placed in subgroup IIc. Three plants from the Rondônia germplasm bank (BAG29, BAG30, and BAG32), which had an intervarietal hybrid passport, were also grouped in Group II, suggesting they belong to the Robusta botanical variety.

Group III consisted primarily of Conilon coffee plants from Embrapa Rondônia’s germplasm bank (Con1089, Con795, Con566, Con154, Con4650, Con201, Con530, Con69, Con556, and Con796), along with BRS Ouro Preto coffee plants (BRSOuroPreto73, BRSOuroPreto61, BRSOuroPreto88, BRSOuroPreto89, BRSOuroPreto160, and BRSOuroPreto57), which also belong to this botanical variety.

The last and largest group (Group V) comprised hybrid coffee plants obtained through open pollination, with the vast majority selected in the field by local farmers. Subgroup Ve contains a concentration of coffee plants that start with the prefix LB (LB22, LB88, LB68, LB33, LB020, LB102, LB12, LB110, LB60, LB15, and LB010), all sourced from a single producer’s field.

Nine F1 hybrids, resulting from artificial crossings between Conilon and Robusta coffee plants, were included in the analysis. Of these, eight hybrids—BAG19, BAG21, BRS3220, BRS1216, BAG26, BRS3213, BAG24, and BRS3210—were grouped into subgroup Vf. The other confirmed F1 hybrid, BRS2314, was placed in subgroup Vh. Additionally, subgroup Vh includes other Embrapa cultivars obtained through open pollination: BRS3193, BRS2299, BRSOuroPreto203, BRSOuroPreto130, BRSOuroPreto184, and BRSOuroPreto155, suggesting that these cultivars are intervarietal hybrids.

An analysis of the 10 BRS cultivars released by Embrapa Rondônia reveals that only the cultivar BRS2357 was allocated to Group III. This cultivar is the sole representative from the Conilon varietal group, while the others were released as intervarietal hybrids, as confirmed by molecular data. These hybrid cultivars are classified in Group V and distributed across subgroup Vf (BRS3220, BRS1216, BRS3213, and BRS3210), subgroup Vg (BRS3137 and BRS2336), and subgroup Vh (BRS2299, BRS3193, and BRS2314).

**Figure 2:**
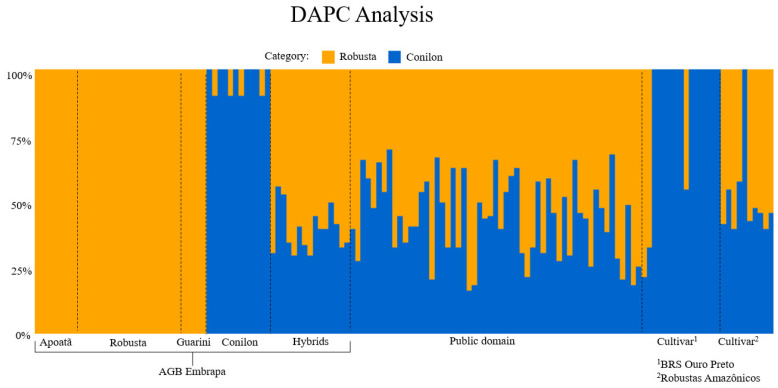
Discriminant Analysis of Principal Components (DAPC) based on SNP data from *Coffea canephora* genotypes. Each vertical bar represents an individual, with color proportions indicating posterior membership probabilities to two genetic clusters: Robusta (orange) and Conilon (blue). Genotypes are grouped by category: Apoatã, Robusta, Guarini, Conilon, and Hybrids (AGB Embrapa), public domain, and cultivars (^1^ BRS Ouro Preto and ^2^ Robustas Amazônicos). The analysis reveals clear genetic differentiation between Robusta and Conilon backgrounds, while hybrids and public domain genotypes show varying degrees of admixture.

The Discriminant Analysis of Principal Components (DAPC) was used to assess the genetic structure of the *C. canephora* genotypes based on SNP data. The bar plot displays the posterior membership probabilities of each genotype to two inferred genetic clusters, designated here as Robusta (orange) and Conilon (blue). Each vertical bar represents a genotype, and the color proportion within each bar indicates the probability of assignment to either cluster (Figure 2).

The results reveal a clear genetic differentiation between groups traditionally associated with Robusta and Conilon backgrounds. Genotypes classified under the Apoatã and Robusta categories (AGB Embrapa collection) show near-complete membership to the Robusta cluster, whereas genotypes labeled as Conilon are predominantly assigned to the Conilon cluster, confirming the consistency between traditional classification and molecular-based structure.

Interestingly, the hybrids exhibit varying degrees of admixture, as expected, with individuals showing intermediate membership probabilities to both clusters. This supports the idea that these hybrids carry alleles from both genetic backgrounds and may harbor useful heterotic potential for breeding programs.

Public domain genotypes also demonstrate substantial admixture, with diverse genetic contributions from both clusters. This pattern suggests that these genotypes may result from historical crosses or introgressions between Robusta and Conilon, reflecting the complexity of breeding histories and gene flow in this germplasm.

The two cultivar groups, BRS Ouro Preto and Robustas Amazônicos, also exhibit distinct patterns. While the former is predominantly assigned to the Robusta cluster, the latter includes individuals with mixed ancestry, indicating either a broader genetic base or potential intervarietal crosses in their development.

Overall, the DAPC results support the presence of two major genetic groups within the dataset and highlight the usefulness of this method for visualizing population structure and identifying admixed individuals. These findings are particularly valuable for guiding crossing strategies, preserving genetic diversity, and optimizing the use of hybrid vigor in *C. canephora* breeding programs.

### 2.2. Segregation of Resistance Markers

Molecular markers associated with QTL-GL2 (linkage group 2) and QTL-GL5 (linkage group 5) were accessed to verify the presence of resistance genes against *H. vastatrix*. QTLs are part of the genetic map constructed by Pestana et al. (2015) [36] and confer resistance to races I, II, and pathotype 001 of *H. vastatrix*.

The analysis of locus A was conducted using the SSR016 marker linked to QTL-GL2 [24,26]. This marker displays a codominant banding pattern, allowing for the identification of homozygous and heterozygous individuals (AA, Aa, and aa). As a result, 47 coffee plants exhibited the resistance allele of QTL-GL2 in dominant homozygosity (AA), 41 in heterozygosity (Aa), and 22 in recessive homozygosity (aa) (Table 1).

Analyzing the CaRHv9 marker, which is linked to QTL-GL5 (Locus B), it was found that 51 coffee plants exhibited the resistance allele. This marker displays a pattern of dominant bands, making it impossible to distinguish between homozygous and heterozygous individuals. As a result, individuals carrying the marker were assigned the genotype B_.

It was observed that most of the analyzed accessions possessed the resistance allele at either locus A, locus B, or both. The presence of the resistance allele at only one locus can provide field resistance, depending on the occurrence of *H. vastatrix* pathogens in the region. In contrast, having the resistance allele at both loci, as seen in 45 coffee plants, may confer more durable resistance to the pathogen. This result highlights the potential of these coffee plants from Rondônia as a source of genes that confer resistance to races I and II, as well as pathotype 001 of *H. vastatrix*.

A fourth locus associated with resistance to *H. vastatrix* was also analyzed (Locus C). The dominant marker CARF005 is derived from expressed sequence tags (ESTs) and was previously confirmed by Alvarenga et al. [25] through the amplification of a DNA region corresponding to a partial open reading frame (ORF) of the *C. arabica* genome. Barka et al. [28], examining a BAC library constructed from the rust-resistant clone of the Timor Hybrid CIFC 832/2, identified and characterized the NB-ARC gene, monitored by the CARF005 marker, as a new resistance gene. This gene was located on the genetic map of *C. arabica* at a locus distinct from QTL-GL2 and QTL-GL5 [25,28].

Among the 110 coffee plants evaluated, 82 were found to carry the resistance gene marked by CARF005 (C_). Since this is a dominant marker, it is not possible to distinguish between heterozygous and homozygous dominant coffee plants.

To pyramid the maximum number of alleles conferring resistance to *H. vastatrix*, a fifth locus was also analyzed (Locus D) using the RLK2 marker. Almeida et al. [24] developed a functional molecular marker called RLK2, which monitors the gene HdT_LRR_RLK2. This gene is a potential new resistance gene not found in differentiating coffee plants. In their study, Almeida et al. [24] observed that this gene exhibited differential expression in the resistant genotype. Consequently, the RLK2 marker is dominant and capable of distinguishing between resistant and susceptible coffee plants at this locus.

As a dominant marker, it allowed for the identification of coffee plants with genotypes D_ and dd. Among the 110 coffee plants analyzed, 33 exhibited the resistance locus, possessing the genotype D_.

Regarding the rust resistance markers, genotypes were identified that exhibited from zero (aa, bb, cc, dd) to four resistance markers (A_, B_, C_, D_). Eighteen genotypes displayed four pyramided resistance markers: N12, N1, N16, BAG30, Rob36, BAG89, Con154, Con530, Con69, SK244, N13, WP6, GB7, P42, AR106, LB10, BAG26, and BAG34. These clones carrying all resistance alleles for rust resistance are distributed across different groups and subgroups of genetic diversity: IIa (BAG30), IId (Rob336), IIIa (BAG89), IIIb (Con154), IIId (Con530, Con89), Vb (N12, N13, SK244), Vc (WP6, GB7), Vd (N1, N16, P42, AR106), Ve (LB10), Vf (BAG26), and Vg (BAG34) (Figure 1).

A larger number of genotypes showed two or three resistance markers, with 39 genotypes having two and 28 genotypes having three markers. In contrast, nineteen genotypes presented one resistance marker, while six genotypes did not exhibit any resistance markers: AP5, BRS2357, AP8, AP1, AP3, and BRS1216.

To track the resistance loci against another important disease, coffee berry disease (CBD), two SSR markers, Sat207 and Sat235, were employed. These markers flank the Ck-1 CBD resistance locus [23,31]. Twenty-four genotypes were identified as carrying resistance alleles at both analyzed markers; 23 were homozygous and one heterozygous (Table 1). The joint analysis of the two markers linked to the Ck-1 resistant allele revealed that some clones lacked one of the markers, indicating the occurrence of recombination events. Thirty genotypes exhibited the resistance marker Sat235 but did not display the marker Sat207. In contrast, twenty-two genotypes showed the marker Sat207 while lacking the marker Sat235. To avoid selecting recombinant plants that may have lost the resistance allele, coffee clones carrying only one marker were not considered to possess the resistance allele.

The genotypes N12, N1, and N16 exhibited pyramiding for all five resistance alleles, comprising four for rust resistance and one for CBD resistance. All of them belong to genetic group V, with N12 assigned to subgroup Vb and N16 and N1 to subgroup Vd. Clones N16 and N1 are genetically very closely related (Figure 1).

**Figure 3:**
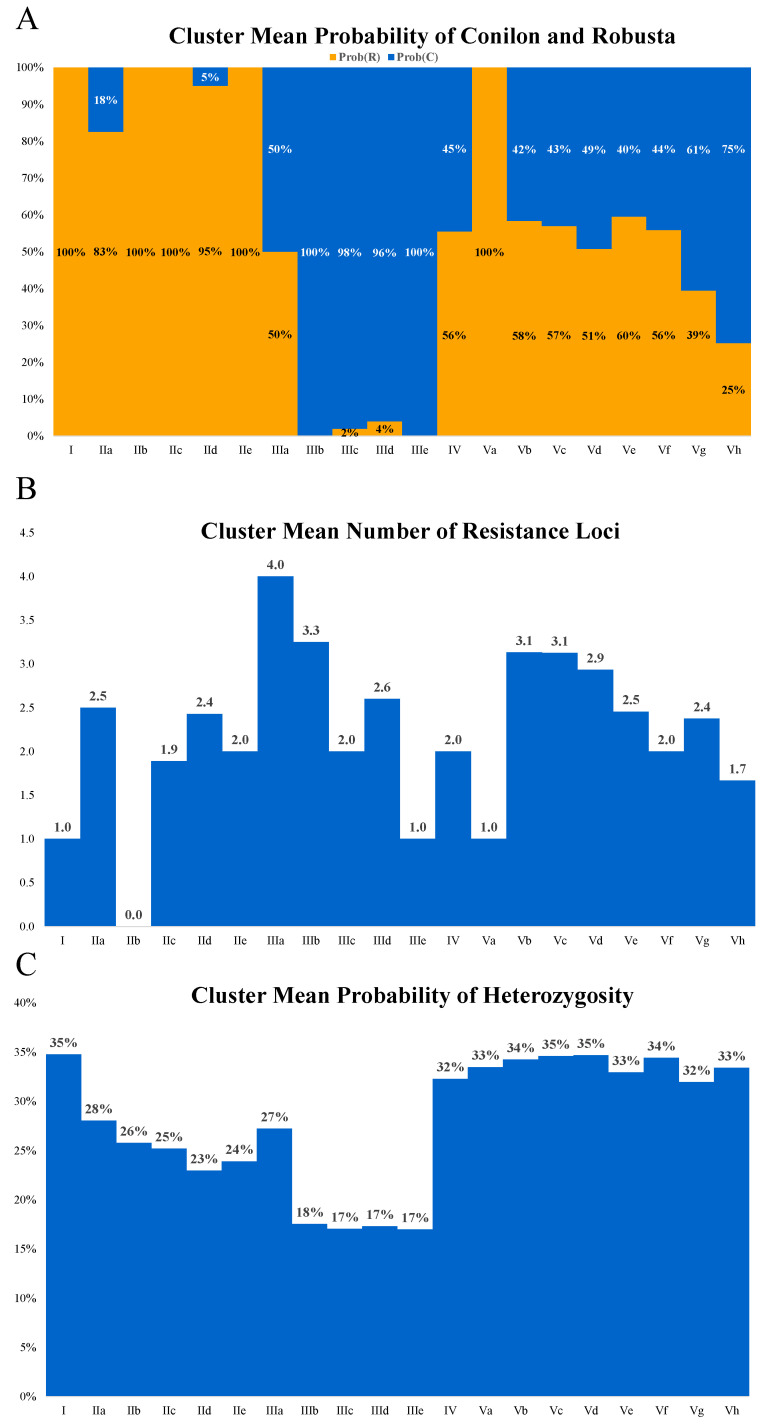
Mean probability of polymorphism origin classified as Conilon or Robusta per group (**A**). Mean probability of heterozygosity per group (**B**). Mean number of rust resistance genes per group (**C**).

The genotypes were organized into distinct genetic clusters, represented by Roman numerals in the dendrogram (I, II, III, IV, and V). The analysis of polymorphism origin revealed clear contrasts among clusters (Figure 3A). Clusters I and II showed a stronger contribution from Robusta, whereas cluster III was predominantly Conilon. Clusters IV and V comprise genotypes carrying polymorphisms from both botanical varieties, reflecting intermediate genetic backgrounds. Among the Conilon-predominant subclusters, IIc and IIIc exhibited probabilities above 60%, reinforcing their closer affinity with Conilon germplasm. Conversely, in addition to cluster I, subgroups such as IIId and IV displayed high proportions of Robusta alleles, in some cases exceeding 70%, confirming their strong association with this variety. A balanced contribution from both origins was observed in cluster IIIa (50% Conilon and 50% Robusta), highlighting not only the differentiation among clusters but also the presence of hybrid patterns in specific subgroups.

The mean probability of heterozygosity also varied substantially among clusters (Figure 3B). The lowest values were detected in subgroups IIIb, IIId, and IIIe, which presented only 17–18% heterozygosity, while clusters IV, Vc, Vd, and Ve showed considerably higher levels, ranging from 34% to 38%. The evaluation of the mean number of rust resistance genes (Figure 3C) revealed additional differences. Cluster IIIa harbored up to four resistance loci, whereas groups such as IIb and Ve averaged three loci. In contrast, some clusters, including IIc, exhibited null values, indicating the absence of detectable resistance alleles. Together, these results underscore the genetic structuring of the population.

## 3. Discussion

The genetic diversity of coffee plants cultivated in Western Amazonia was comprehensively analyzed using molecular markers. This analysis, which included 140 genotypes and 39,329 SNP molecular markers, enabled the classification of coffee plants based on their origin, botanical variety, and genealogy, distinguishing those derived from open pollination from those produced through directed pollination.

Among these genotypes, some clones were selected by the coffee growers themselves, with some being intensively cultivated [35]. However, the pedigree of these clones remains uncertain, as it is not known to which botanical variety they belong or from which they derive the greatest genomic contribution. For instance, clone C25 is believed to be a Robusta coffee plant, as it is included in group I and is genetically closer to the genotypes in group II, which comprises the Robusta group. Among the producers’ clones, GJ2 and GJ20 were the most distinct and divergent from the other genotypes, forming a separate group (group IV) (Figure 1).

Our results confirm the hybrid nature of the Western Amazon coffee cultivation. In the largest group (Group V), hybrid coffee plants obtained through open pollination are grouped, with a predominance of genotypes selected in the field by local farmers.

This genetic diversity can be leveraged both for plant adaptation to different regions and in the context of climate changes and variations. The gradual increase in temperature and irregular rainfall patterns may negatively impact coffee cultivation, leading to yield losses, increased incidence of diseases, and compromising the viability of most growing areas [37]. The lower altitudes and higher temperatures typical of the tropical Amazon climate define the environment where these plants were selected. The consistent performance of these genotypes has also been reported in other environments [10]. While the BRS1216 and BRS2336 cultivars have shown good performance in crops grown in Amazonas, Acre, and Roraima, hybrid clones such as R22, GJ8, GJ25, AS2, and LB15 have been transferred from the Northern region to the Southeast, being cultivated on a large scale [6].

The multiclonal cultivar BRS Ouro Preto, composed of 15 genotypes, is characterized by traits of the Conilon botanical variety [18]. In the present study, thirteen clones from this cultivar were characterized, with six grouped with the Conilons (group III) and seven grouped in the large group V, where the hybrid coffee plants are found, indicating the hybrid nature of these plants within the cultivar.

Genetic diversity data also suggest that the following genotypes maintained in the collection, despite having distinct identifications, may be the same clone or closely related materials: RobGuarini2107 and RobGuarini2106; AP3 and AP2; Rob32 and Rob126; BAG26 and BRS3213.

In addition to enabling selection gains, the greater genetic variability in this coffee species is linked to both the expression of hybrid vigor and the complementary traits of the Conilon and Robusta botanical varieties [38,39]. In the dendrogram, individuals assigned to different groups exhibiting higher genetic divergence may be selected for hybridization. Individuals in groups IIa, IIb, IIc, IId, and IIe, which are characterized by a higher frequency of genotypes from the Robusta botanical variety, have greater potential for hybridization with individuals from Conilon grouped in IIIa, IIIb, IIIc, and IIIe. High-performing hybrid progenies can be obtained from the most genetically divergent plants identified in this study, maximizing the potential for hybrid vigor expression and leveraging the complementary traits of these botanical varieties.

The DAPC analysis identified two main genetic clusters corresponding to the traditionally recognized Robusta and Conilon groups, confirming the effectiveness of SNP markers in elucidating genetic structure. From a breeding perspective, these findings provide valuable insights into the use of genetic variability and the development of new cultivars. The registered cultivars, BRS Ouro Preto and Robustas Amazônicos, showed distinct genetic profiles. BRS Ouro Preto was primarily assigned to the Conilon cluster, consistent with its origin, while Robustas Amazônicos exhibited a higher level of genetic admixture, reflecting a broader genetic foundation shaped by targeted intervarietal crosses.

Additionally, the analysis successfully identified key clones within their respective cultivar groups, such as clone BRSOuroPreto199 in BRS Ouro Preto and clone BRS2357 within Robustas Amazônicos. These findings confirm the consistency between field observations and molecular profiles, underscoring the utility of molecular markers in characterizing breeding materials.

Genotypes classified as public domain, located near the center of the DAPC plot, exhibited significant Robusta ancestry. This pattern suggests a history of well-documented introgression between Conilon and Robusta in the Amazon region [35]. Furthermore, the AGB Embrapa collection displayed substantial genetic variability, including highly differentiated genotypes. This diversity provides a strong foundation for identifying contrasting parental lines and optimizing crossing strategies.

Although *C. canephora* is a species that carries resistance genes, studies have shown that there are varying levels of resistance among different genotypes, with some coffee plants being resistant, moderately resistant, or even susceptible [34].

Coffee rust, caused by *H. vastatrix*, can be found in nearly all coffee-growing regions and can lead to significant production losses [33,40,41]. At least nine dominant genes present in different coffee species have been characterized, ranging from S_H_1 to S_H_9. The resistance genes S_H_1, S_H_2, S_H_4, and S_H_5 are derived from *C. arabica*, S_H_3 from *C. liberica*, while the genes S_H_6, S_H_7, S_H_8, and S_H_9 have been identified in *C. canephora*. These genes are also present in the Timor Hybrid (HdT), a natural hybrid between *C. arabica* and *C. canephora*. Recently, Barka et al. [28] and Almeida et al. [24] identified and characterized two candidate SH resistance genes to *H. vastatrix* in the Timor Hybrid.

The presence of other resistance alleles and loci that confer coffee plants’ resistance to rust has also been studied in the coffee collection from Rondônia [25,26,42]. The more resistance genes a parent plant possesses, the greater the likelihood of obtaining resistant progenies. Field resistance is also linked to the emergence of new races of *H. vastatrix*, which can evolve more rapidly than the response of a breeding program. The high variability of the pathogen poses a risk to the durable resistance of coffee plants in the field, while this variability also serves as an important source of resistance [24].

Although predicting field resistance responses using molecular markers has limitations due to the complexity of factors influencing resistance, the segregation results of resistance markers are valuable for hybridization and the development of new breeding populations.

Limitations in this prediction can be observed in the group of genotypes that did not exhibit resistance markers for rust. While the clone BRS2357 is characterized by its susceptibility to rust, the genotypes of the Apoatã botanical variety (AP5, AP8, AP1, AP3) and the cultivar BRS1216 are known in the field for their high resistance to this pathogen. However, in the context of producing resistant progenies, hybridizing parent plants with a higher frequency of resistance markers increases the likelihood of pyramiding these genes and obtaining resistant plants from previously characterized parents.

Among the more than 50 races of *H. vastatrix* identified, race II stands out as the most common and widely distributed in the country [33]. Molecular data for QTL/GL2 (Locus A) indicated 47 homozygous resistant coffee plants (AA), 42 heterozygous resistant plants (Aa), and 22 homozygous susceptible plants (aa). For QTL/GL5 (Locus B), the marker used displayed a dominant pattern, allowing the identification of 51 resistant coffee plants (B_).

The resistance of coffee plants to races I, II, and pathotype 001 of *H. vastatrix* is conferred by two independent dominant loci [43]. Therefore, the presence of a dominant allele at either locus indicates a higher frequency of resistance alleles [24]. The best sources of resistance correspond to coffee plants with the genotype AAB_, as they possess both genes, leading to greater durability of resistance and homozygosity at locus A. For locus B, it was not possible to distinguish between homozygous and heterozygous plants, as the available molecular marker exhibits dominant behavior. The coffee plants with the genotype AAB_ include BAG 34, BAG 33, BAG 30, N1, AS5, Rob128, Rob36, GB4, WP6, Con530, Con69, LB60, LB88, LB110, and LB160.

The marker CARF005 (Locus C) enabled the identification of 82 resistant coffee plants. A significant number of coffee plants exhibited this dominant resistance marker, which may be attributed to the fact that this gene was cloned from Timor Hybrids that possess resistance genes from the species *C. canephora* [28].

The other gene previously cloned and monitored in this study corresponds to Locus D. With a dominant profile, this marker allowed for the identification of 33 coffee plants containing the resistance marker. The marker RLK2 was developed based on the presence and absence of the gene HdT_LRR_RLK2 in various differential coffee clones. These clones contain different combinations of rust resistance genes. Almeida et al. [24] noted that the gene HdT_LRR_RLK2 does not correspond to any of the previously characterized resistance genes (SH 1-9).

Coffee plants with the other four pyramided resistance genes were also identified, constituting promising sources of resistance. Notably, the coffee plants BAG30, Rob36, Con530, Con69, WP6, N1, P42, LB10, and BAG34 contain Locus A in dominant homozygosity, which means it will not segregate in the field. These coffee plants are potential parents for breeding programs, as they possess four pyramided rust resistance alleles.

Molecular markers were also employed to monitor loci of resistance to another disease known as CBD, caused by the fungus Colletotrichum kahawae. Two SSR markers were used, both identified and mapped by Gichuru et al. [31] and validated by Alkimin et al. [23]. These markers flank the gene Ck-1, which confers resistance to the disease. Through the combined analysis of the markers, 24 homozygous resistant coffee plants were identified. These individuals exhibit resistance markers for both molecular markers (Sat207 and Sat235). The observed recombinations—meaning the presence of only one marker—can also lead to the loss of resistance genes [44]. This is due to the estimated distance between the two markers, which is 17.2 cM [31]. The greater the distance between markers, the higher the chance of recombination occurring [29].

Although CBD is a disease restricted to the African continent, preventive breeding has proven important in coffee cultivation. In the 1970s, anticipating the arrival of rust in Brazil, resistant cultivars of *C. arabica* were evaluated in other countries, facilitating better management of this disease at the time. The availability of these markers increases the likelihood of obtaining resistant progenies even in the absence of the pathogen [23,27,41].

In the characterization of marker segregation, it was possible to identify coffee plants containing the highest number of pyramided alleles for both diseases. The coffee plants N1, N12, and N16 carry the four resistance alleles for rust evaluated in this study and also possess the allele for CBD (A_B_C_D_EE). These three coffee plants are open-pollinated hybrids selected by the same producer (Table 2). The dendrogram also shows that the coffee plants N1 and N16 are genetically similar, grouped in subgroup Vd.

Divergent crosses have shown promising results in obtaining plants with greater vigor and productivity, as well as exhibiting complementary characteristics of the botanical varieties Conilon and Robusta. The results of this study allow for the selection of matrices with greater potential for hybrid progeny production, based on the selection of matrices from different botanical varieties that carry resistance genes and are grouped in different clusters. For example, one can perform a cross between the coffee plant identified as Con154, which possesses pyramided genes (AaB_C_D_) and is allocated in group IIIb of the dendrogram, with the coffee plant identified as Rob36 (AAB_C_D_ee). This plant also has pyramided rust resistance genes and is allocated in linkage group IId.

Coffee cultivation has been part of the Western Amazon landscape since its colonization in the mid-1970s [36]. However, significant production highlights were achieved years after the cultivation began [6]. Currently, this region is the second-largest national producer of *C. canephora* [45,46]. Although coffee is part of a robust and structured production chain, it faces challenges such as global climate change [32,37]. Climate change may pose risks to resistance against both biotic and abiotic stresses. For example, studies project a reduction in the incubation period of coffee leaf rust, which could result in more severe epidemics [1].

The results of this study demonstrate the hybrid nature of the plants cultivated in Rondônia, a coffee culture carried out in the tropics characterized by low altitudes, high temperatures, and acidic soils, which favor the selection of plants with greater resilience to climate change.

The use of molecular markers in breeding programs allows for advancements in obtaining new breeding populations, assisting breeders in their decision-making processes.

## 4. Materials and Methods

### 4.1. Genetic Materials

A total of 140 coffee plants of the species *C. canephora* were genotyped on a large scale using SNP markers. These genotypes are sourced from the breeding program of the Brazilian Agricultural Research Corporation (Embrapa, Rondônia, Brazil), along with genotypes from local producers. Among the genotypes, there are coffee plants from the botanical varieties Conilon and Robusta, as well as hybrids (Table 2).

Leaf samples were collected separately and lyophilized for shipment to the Coffee Biotechnology Laboratory—BioCafé at the Federal University of Viçosa, Minas Gerais, Brazil. Genomic DNA extraction was performed using the method proposed by Diniz et al. [35]. The quality of the DNA was assessed on an agarose gel, and quantification was conducted using a NanoDrop 2000 (Thermo Fisher Scientific, Waltham, MA, USA). The DNA concentration of the samples was standardized and sent to Rapid Genomics in Florida, USA, for sequencing and identification of SNP molecular markers.

Sequencing-based genotyping was conducted using targeted enrichment followed by Next-Generation Sequencing (NGS) [47]. A total of 140 *C. canephora* samples were prepared for NGS and hybridized with 10,000 solution-synthesized probes. These probes were designed using reference sequences from the Brazilian Coffee Genome Project and the *C. canephora* genome database [22,39]. Sequencing was performed on an Illumina Hi-Seq platform, and SNP markers were identified through bioinformatics analysis (RAPiD Genomics, Gainesville, FL, USA).

### 4.2. Quality Analysis of SNP Markers

SNP calling was carried out by RAPiD Genomics (Gainesville, FL, USA), with sequence reads aligned to the *Coffea canephora* reference genome (DHA84/CR genotype) [45]. The resulting SNP dataset was filtered using the following quality parameters: MinDP = 3, DP range = 15–750, missing rate ≤ 0.4, MAF ≥ 0.01, MinQ = 10, and call rate ≥ 0.95. Quality control analyses were conducted in RStudio version 4.2.1, and SNPs not meeting these thresholds were excluded from the dataset.

MinDP3 refers to the minimum sequencing depth per SNP and per individual. SNPs with values lower than this parameter were considered as missing data. DPrange denotes the average sequencing depth range of individuals for which a SNP is retained. SNP markers with average coverage in individuals outside the specified range for each filter were removed. Miss represents the percentage of missing data accepted in individuals after filtering by the MinDP parameter. SNP markers with missing values exceeding the defined threshold were eliminated. MAF is a measure related to allele variation in the population, where lower-frequency alleles are discarded due to their limited informativeness and lack of genetic relevance in individuals. Call Rate is a metric used to eliminate SNPs with a high number of missing values, while MinQ indicates the minimum quality accepted for SNPs. Therefore, SNPs with quality values below the thresholds established by the quality criteria used in this study were also removed.

### 4.3. Genetic Diversity Analysis

For the genetic diversity analysis, SNP data were coded as 0, 1, and 2. Since these are biallelic and codominant markers, 0 indicates individuals homozygous for the less frequent allele, 1 indicates heterozygous individuals, and 2 indicates individuals homozygous for the more frequent allele. Thus, coffee plants with genotypes A_1_A_1_, A_1_A_2_, or A_2_A_2_ were coded as 11, 12, and 22, respectively.

Genetic diversity was assessed using a genetic dissimilarity matrix calculated with Nei’s dissimilarity coefficients [48]. The matrix was generated using the Nei.dist function from the Poppr package, and a hierarchical clustering was subsequently performed using the Hclust function with the Unweighted Pair Group Method with Arithmetic Mean (UPGMA). The dendrogram was obtained using the Fviz-dend function from the FactoExtra package. All analyses were conducted in the RStudio environment, version 4.2.1 (Figure 1).

Discriminant Analysis of Principal Components (DAPC) was employed to investigate the genetic structure among genotypes. This method combines Principal Component Analysis (PCA) and Discriminant Analysis (DA) to summarize genetic differentiation between predefined groups while minimizing within-group variation. First, PCA was used to transform the original SNP dataset into a set of uncorrelated principal components, effectively reducing dimensionality and eliminating multicollinearity. Then, DA was performed on the retained principal components to maximize the separation between groups. Unlike model-based approaches such as STRUCTURE or ADMIXTURE, DAPC does not rely on assumptions of Hardy–Weinberg equilibrium or linkage disequilibrium, making it well suited for datasets with complex breeding histories or admixed individuals. The number of clusters was set to two, based on prior knowledge of the germplasm, which reflects the distinction between the two botanical varieties, Conilon and Robusta [13,49]. The analysis provided, for each genotype, the posterior probability of membership to each group, allowing the assessment of genetic clustering and potential admixture. DAPC was conducted using the adegenet package in R [50] (Figure 2).

The cluster mean probability of Conilon and Robusta gene origin was inferred from DAPC (Discriminant Analysis of Principal Components), while mean heterozygosity was estimated from SNP genotype distribution (Figure 3).

### 4.4. Molecular Markers Associated with Resistance to Rust and CBD

Of the 140 coffee plants analyzed in the molecular genetic diversity study, a sample of 110 was selected to identify the resistance alleles present at different loci using molecular markers. These coffee plants were analyzed with molecular markers linked to genes that confer resistance to the two main fungal diseases affecting coffee: rust caused by the fungus *Hemileia vastatrix* and coffee berry disease (CBD) caused by *Colletotrichum kahawae* (Table 3).

The purified DNA from the coffee plants was amplified using six molecular markers previously identified as being associated with genes conferring resistance to *H. vastatrix* and *C. kahawae* (Table 3). Four loci associated with resistance to *H. vastatrix* were analyzed: QTL-GL2, named Locus A, monitored by the marker SSR016 [26]; QTL-GL5, named Locus B, monitored by the marker CaRHv9 [24]; the candidate gene NB-ARC, Locus C, monitored by the marker CARF005 [28]; and Locus D, for another candidate resistance gene HdT_LRR_RLK2, monitored by the marker RLK2 [24]. For the analysis of resistance to *C. kahawae*, the Ck-1 gene, named Locus E, was evaluated. This gene was monitored by two markers flanking the gene, Sat235 and Sat207 [23,31].

### 4.5. Markers SSR016 and CaRHv9

Locus A corresponds to a QTL (Quantitative Trait Locus) for resistance to races I, II, and pathotype 001 of *H. vastatrix*, located on linkage group 2 (LG2) of the genetic map and monitored by the codominant marker SSR016 [26]. Locus B corresponds to a second QTL for resistance to the same pathogens, located on linkage group 5 (LG5) and associated with the dominant marker CaRHv9 (Table 3). The resistant genotype Timor Hybrid UFV 443-03 and the susceptible Catuaí Amarelo IAC 64 were used as controls.

For SSR016, PCR amplifications were carried out in 20 μL reactions containing 50 ng of genomic DNA, 1X PCR buffer, 1.0 mM MgCl_2_, 0.15 mM of each dNTP, 0.1 μM of each primer, and 0.6 U of Taq polymerase (Invitrogen, Waltham, MA, USA). The touchdown PCR program consisted of an initial denaturation at 94 °C for 2 min; 10 cycles of 94 °C for 30 s, annealing at 66 °C for 30 s (decreasing by 1 °C per cycle), and 72 °C for 30 s; followed by 30 cycles at 94 °C, 57 °C, and 72 °C for 30 s each. A final extension was performed at 72 °C for 20 min. Genotyping was conducted by capillary electrophoresis on an ABI 3130xl Genetic Analyzer (Applied Biosystems, Waltham, MA, USA).

For CaRHv9, PCR reactions were performed in 20 μL containing 50 ng of DNA, 1X PCR buffer, 2.0 mM MgCl_2_, 0.15 mM of each dNTP, 0.1 μM of each primer, and 1 U of Taq polymerase (Invitrogen, Waltham, MA, USA). Cycling conditions were 94 °C for 5 min; 32 cycles of 94 °C for 30 s, 65 °C for 30 s, and 72 °C for 1 min; and a final extension at 72 °C for 10 min. PCR products were analyzed on 1.5% agarose gels and visualized with ethidium bromide (0.5 μg·mL^−1^).

### 4.6. Marker CARF005

Locus C corresponds to the dominant marker CARF005, derived from RGAs (Resistance Gene Analogs), which amplifies a DNA region associated with proteins mediating rust resistance in coffee [25,28]. The resistant genotype Timor Hybrid CIFC 832/2 and the susceptible Caturra Vermelho CIFC 19/1 were used as controls.

PCR was performed in 20 μL reactions containing 5 ng of DNA, 1X PCR buffer, 1 mM MgCl_2_, 0.2 mM dNTPs, 0.2 μM primers, and 0.8 U of Taq polymerase (Invitrogen). The program included 95 °C for 5 min; 35 cycles of 94 °C for 30 s, 60 °C for 30 s, and 72 °C for 1 min; and a final extension at 72 °C for 10 min. PCR products were resolved on 1.5% agarose gels and stained with ethidium bromide (0.5 μg·mL^−1^).

### 4.7. Marker RLK

Locus D corresponds to the functional marker RLK2, developed by Almeida et al. [24] from the HdT_LRR_RLK2 gene identified in Timor Hybrid CIFC 832/2. The resistant genotype Timor Hybrid CIFC 832/1 and the susceptible Caturra Vermelho CIFC 19/1 were used as controls.

PCR was performed in 20 μL reactions containing 50 ng of DNA, 1X PCR buffer, 1 mM MgCl_2_, 0.15 mM dNTPs, 0.1 μM primers, and 1 U of Taq polymerase (Invitrogen). Cycling conditions were 94 °C for 5 min; 34 cycles of 94 °C for 30 s, 66 °C for 30 s, and 72 °C for 1 min; and a final extension at 72 °C for 10 min. Genotyping was performed by capillary electrophoresis on an ABI 3130xl Genetic Analyzer (Applied Biosystems).

### 4.8. Markers Sat207 and Sat235

Loci E and F correspond to the dominant and epistatic markers Sat207 and Sat235, linked to the Ck-1 gene. These markers were identified and mapped by Gichuru et al. [9] and later used by Alkimim et al. [23]. The resistant genotypes Timor Hybrids UFV 377-15 and UFV 440-10 and the cultivar MGS Catiguá 3 were used as controls, along with the susceptible genotypes Caturra Vermelho CIFC 19/1 and Catuaí Vermelho IAC 64 (UFV 2148-57).

PCR amplifications were conducted in 25 μL reactions containing 50 ng of DNA, 1X PCR buffer, 2.0 mM MgCl_2_, 0.1 mM dNTPs, 0.4 μM primers, and 0.5 U of Taq polymerase (Invitrogen). The program consisted of 95 °C for 5 min; 35 cycles of 94 °C for 45 s, 50 °C for 45 s, and 72 °C for 45 s; and a final extension at 72 °C for 10 min. Genotyping was performed by capillary electrophoresis on an ABI 3130xl Genetic Analyzer (Applied Biosystems).

## 5. Conclusions

Molecular SNP markers proved effective in assessing genetic diversity. Coffee plants from the Robusta botanical variety are grouped in Cluster II, those from the Conilon variety in Cluster III, and the intervarietal hybrids in Cluster V. The DAPC results revealed significant genetic patterns within breeding materials and cultivated clones. These findings are important for guiding selection decisions and understanding the genetics of the cultivated clones. Although molecular characterization for resistance to coffee leaf rust and coffee berry disease (CBD) does not fully predict field resistance responses, it enabled the identification of coffee plants harboring various resistance genes for these diseases. With the goal of developing clones and hybrids exhibiting multiple resistances to both rust and CBD and resilient to climate change, these results provide valuable direction for future crosses in coffee breeding programs.

## Figures and Tables

**Table 1:** Molecular screening for coffee resistance to rust in QTL for resistance to races I, II, and pathotype 001 (Loci A and B); NB-ARC (Locus C); HdT_LRR_RLK2 (Locus D); and Ck-1 for CBD (markers E and F). Ck1-1 and Ck1-2 correspond to flanking markers of the same locus.

Gen.	QTLGL2	QTLGL10	NB-ARC	RLK2	Ck1-1	Ck1-2	R. loci ^a^	Gen.	QTLGL2	QTLGL10	NB-ARC	RLK2	Ck1-1	Ck-1-2	R. loci
N12	Aa	B_	C_	D_	EE	FF	5	BRS2299	Aa	B_	cc	dd	EE	FF	3
N1	AA	B_	C_	D_	EE	FF	5	Rob211	aa	bb	C_	D_	ee	ff	2
N16	Aa	B_	C_	D_	EE	FF	5	AP2	Aa	bb	cc	dd	EE	FF	2
BAG30	AA	B_	C_	D_	ee	FF	4	Rob272	AA	bb	C_	dd	EE	ff	2
Rob36	AA	B_	C_	D_	ee	FF	4	Rob32	Aa	bb	C_	dd	EE	ff	2
BAG89	Aa	B_	C_	D_	ee	FF	4	Rob126	Aa	bb	cc	D_	EE	ff	2
Con154	Aa	B_	C_	D_	ee	ff	4	Rob63	aa	bb	C_	D_	ee	FF	2
Con530	AA	B_	C_	D_	EE	ff	4	Rob65	Aa	bb	cc	dd	EE	FF	2
Con69	AA	B_	C_	D_	ee	ff	4	Rob66	AA	bb	C_	dd	EE	ff	2
SK244	Aa	B_	C_	D_	ee	Ff	4	Con4650	AA	bb	C_	dd	ee	ff	2
SK41	AA	B_	C_	dd	EE	FF	4	Con201	aa	B_	C_	dd	ee	ff	2
N13	Aa	B_	C_	D_	ee	FF	4	Con556	AA	B_	cc	dd	ee	ff	2
N2	Aa	B_	C_	dd	EE	FF	4	GJ1	Aa	bb	C_	dd	ee	ff	2
WP6	AA	B_	C_	D_	ee	FF	4	N32	Aa	bb	C_	dd	ee	ff	2
LB160	AA	B_	C_	dd	Ee	Ff	4	LB80	aa	B_	C_	dd	ee	ff	2
GB7	Aa	B_	C_	D_	EE	ff	4	L1	AA	bb	C_	dd	ee	FF	2
P42	AA	B_	C_	D_	EE	ff	4	BG180	aa	bb	C_	D_	ee	ff	2
AR106	Aa	B_	C_	D_	ee	FF	4	P50	Aa	B_	cc	dd	EE	ff	2
GJ30	AA	B_	C_	dd	EE	FF	4	AS1	aa	bb	C_	dd	EE	FF	2
LB10	AA	B_	C_	D_	ee	ff	4	AS7	AA	bb	cc	D_	EE	ff	2
BAG26	Aa	B_	C_	D_	ee	ff	4	BAG41	AA	bb	C_	dd	ee	FF	2
BAG34	AA	B_	C_	D_	ee	ff	4	AS10	AA	bb	C_	dd	ee	FF	2
Rob224	Aa	bb	C_	dd	EE	FF	3	GJ5	Aa	bb	C_	dd	ee	FF	2
AP7	Aa	bb	C_	dd	EE	FF	3	LB22	AA	bb	C_	dd	ee	Ff	2
AP6	aa	bb	C_	D_	EE	FF	3	LB33	AA	bb	C_	dd	Ee	ff	2
AP4	Aa	bb	C_	D_	EE	ff	3	LB20	AA	bb	C_	dd	ee	Ff	2
BAG29	Aa	bb	C_	D_	EE	ff	3	LB102	AA	bb	C_	dd	ee	FF	2
Rob128	AA	B_	C_	dd	ee	ff	3	LB12	AA	bb	C_	dd	ee	ff	2
Con1089	AA	B_	C_	dd	ee	FF	3	BAG28	aa	bb	C_	dd	Ee	FF	2
Con795	Aa	B_	C_	dd	ee	ff	3	BRS3213	Aa	bb	C_	dd	ee	FF	2
Con566	AA	B_	C_	dd	ee	FF	3	BAG24	Aa	bb	C_	dd	ee	FF	2
GJ20	aa	B_	C_	dd	EE	FF	3	BRS3210	Aa	bb	C_	dd	ee	ff	2
N7	Aa	B_	C_	dd	ee	FF	3	BAG23	AA	bb	C_	dd	ee	FF	2
VP156	Aa	B_	C_	dd	ee	Ff	3	Con1048	Aa	B_	cc	dd	ee	FF	2
R22	AA	B_	C_	dd	EE	ff	3	RobIAC8	aa	bb	C_	dd	EE	ff	1
AS3	aa	B_	C_	dd	EE	FF	3	BAG32	AA	bb	cc	dd	EE	ff	1
SK80	AA	B_	C_	dd	ee	FF	3	AP5	aa	bb	cc	dd	EE	FF	1
N8(G8)	aa	B_	cc	D_	EE	FF	3	Rob124	Aa	bb	cc	dd	EE	ff	1
GB1	AA	bb	C_	D_	ee	ff	3	Con796	AA	bb	cc	dd	ee	ff	1
GJ21	AA	bb	C_	dd	EE	FF	3	BRS2357	aa	bb	cc	dd	EE	FF	1
GB4	AA	B_	C_	dd	ee	FF	3	GJ2	AA	bb	cc	dd	Ee	ff	1
LB7	Aa	B_	C_	dd	ee	Ff	3	AP8	aa	bb	cc	dd	EE	FF	1
AS5	AA	B_	C_	dd	ee	FF	3	AS12	aa	bb	C_	dd	EE	ff	1
AS6	Aa	B_	C_	dd	ee	FF	3	LB68	aa	bb	C_	dd	ee	ff	1
AS2	Aa	B_	cc	dd	EE	FF	3	GJ8	AA	bb	cc	dd	ee	FF	1
R152	Aa	B_	C_	dd	ee	FF	3	BAG19	Aa	bb	cc	dd	ee	ff	1
LB88	AA	B_	C_	dd	ee	ff	3	BAG21	Aa	bb	cc	dd	ee	FF	1
LB110	AA	B_	C_	dd	ee	Ff	3	BRS3220	Aa	bb	cc	dd	ee	ff	1
LB60	AA	B_	cc	D_	ee	ff	3	BRS3137	aa	bb	C_	dd	EE	ff	1
LB15	AA	B_	C_	dd	EE	ff	3	BRS2336	AA	bb	cc	dd	EE	ff	1
GJ30	Aa	bb	cc	D_	EE	Ff	3	BRS3193	AA	bb	cc	dd	ee	FF	1
GJ25	aa	B_	C_	D_	ee	FF	3	BRS2314	Aa	bb	cc	dd	ee	FF	1
BAG33	AA	B_	C_	dd	ee	FF	3	AP1	aa	bb	cc	dd	ee	ff	0
BAG22	Aa	bb	C_	D_	ee	FF	3	AP3	aa	bb	cc	dd	EE	ff	0
Con854	AA	bb	C_	D_	ee	ff	3	BRS1216	aa	bb	cc	dd	ee	ff	0

^a^ R. loci—number of disease resistance loci. Gen.: genotype.

**Table 2:** Identification of the analyzed coffee genotypes, distinguishing botanical variety, pedigree, and origin. Commercially available genotypes in the public domain, identified by at least the same number, share a common origin.

*n*	Genotype	Field Classification	Genealogy	Origin
1	RobIAC8	Robusta	Open pollination	AGB Embrapa
2	C25	Robusta	Open pollination	AGB Embrapa
3	BAG32	Hybrid	Open pollination	AGB Embrapa
4	BAG30	Hybrid	Open pollination	AGB Embrapa
5	RobIAC6	Robusta	Open pollination	AGB Embrapa
6	RobIAC7	Robusta	Open pollination	AGB Embrapa
7	RobGuarini2102	Robusta	Open pollination	AGB Embrapa
8	RobGuarini2109	Robusta	Open pollination	AGB Embrapa
9	RobGuarini2104	Robusta	Open pollination	AGB Embrapa
10	RobGuarini2107	Robusta	Open pollination	AGB Embrapa
11	RobGuarini2106	Robusta	Open pollination	AGB Embrapa
12	Rob224	Robusta	Open pollination	AGB Embrapa
13	Rob211	Robusta	Open pollination	AGB Embrapa
14	AP7	Robusta	Open pollination	AGB Embrapa
15	AP6	Robusta	Open pollination	AGB Embrapa
16	AP4	Robusta	Open pollination	AGB Embrapa
17	AP5	Robusta	Open pollination	AGB Embrapa
18	AP1	Robusta	Open pollination	AGB Embrapa
19	AP3	Robusta	Open pollination	AGB Embrapa
20	AP2	Robusta	Open pollination	AGB Embrapa
21	BAG29	Hybrid	Open pollination	AGB Embrapa
22	Rob272	Robusta	Open pollination	AGB Embrapa
23	Rob128	Robusta	Open pollination	AGB Embrapa
24	Rob124	Robusta	Open pollination	AGB Embrapa
25	Rob36	Robusta	Open pollination	AGB Embrapa
26	Rob32	Robusta	Open pollination	AGB Embrapa
27	Rob126	Robusta	Open pollination	AGB Embrapa
28	Rob63	Robusta	Open pollination	AGB Embrapa
29	RobIAC1	Robusta	Open pollination	AGB Embrapa
30	Rob65	Robusta	Open pollination	AGB Embrapa
31	Rob66	Robusta	Open pollination	AGB Embrapa
32	RobIAC4	Robusta	Open pollination	AGB Embrapa
33	RobIAC5	Robusta	Open pollination	AGB Embrapa
34	RobIAC3	Robusta	Open pollination	AGB Embrapa
35	RobIAC2	Robusta	Open pollination	AGB Embrapa
36	BAG89	Hybrid	Open pollination	AGB Embrapa
37	Con1089	Conilon	Open pollination	AGB Embrapa
38	Con795	Conilon	Open pollination	AGB Embrapa
39	Con566	Conilon	Open pollination	AGB Embrapa
40	Con154	Conilon	Open pollination	AGB Embrapa
41	BRSOuroPreto73	Conilon	Open pollination	Cultivar
42	BRSOuroPreto61	Conilon	Open pollination	Cultivar
43	Con4650	Conilon	Open pollination	AGB Embrapa
44	BRSOuroPreto88	Conilon	Open pollination	Cultivar
45	BRSOuroPreto89	Conilon	Open pollination	Cultivar
46	Con201	Conilon	Open pollination	AGB Embrapa
47	Con530	Conilon	Open pollination	AGB Embrapa
48	Con69	Conilon	Open pollination	AGB Embrapa
49	Con556	Conilon	Open pollination	AGB Embrapa
50	Con796	Conilon	Open pollination	AGB Embrapa
51	BRSOuroPreto160	Conilon	Open pollination	Cultivar
52	BRSOuroPreto57	Conilon	Open pollination	AGB Embrapa
53	BRS2357	Conilon	Open pollination	Cultivar
54	GJ2	Hybrid	Open pollination	Public domain ^1^
55	GJ20	Hybrid	Open pollination	Public domain ^1^
56	AP8	Robusta	Open pollination	AGB Embrapa
57	SK244	Hybrid	Open pollination	Public domain ^2^
58	GJ1	Hybrid	Open pollination	Public domain ^1^
59	N7	Hybrid	Open pollination	Public domain ^3^
60	CA1	Hybrid	Open pollination	Public domain ^4^
61	N32	Hybrid	Open pollination	Public domain ^3^
62	VP156	Hybrid	Open pollination	Public domain ^5^
63	LB015	Hybrid	Open pollination	Public domain ^6^
64	LB80	Hybrid	Open pollination	Public domain ^6^
65	L1	Hybrid	Open pollination	Public domain ^7^
66	R22	Hybrid	Open pollination	Public domain ^8^
67	AS3	Hybrid	Open pollination	Public domain ^10^
68	SK80	Hybrid	Open pollination	Public domain ^2^
69	SK41	Hybrid	Open pollination	Public domain ^2^
70	N13	Hybrid	Open pollination	Public domain ^3^
71	N12	Hybrid	Open pollination	Public domain ^3^
72	N2	Hybrid	Open pollination	Public domain ^3^
73	N8(G8)	Hybrid	Open pollination	Public domain ^3^
74	AS12	Hybrid	Open pollination	Public domain ^10^
75	GB1	Hybrid	Open pollination	Public domain ^11^
76	GJ21	Hybrid	Open pollination	Public domain ^1^
77	GB4	Hybrid	Open pollination	Public domain ^11^
78	WP6	Hybrid	Open pollination	Public domain ^15^
79	LB160	Hybrid	Open pollination	Public domain ^6^
80	LB7	Hybrid	Open pollination	Public domain ^6^
81	GB7	Hybrid	Open pollination	Public domain ^11^
82	P42	Hybrid	Open pollination	Public domain ^13^
83	R152	Hybrid	Open pollination	Public domain ^9^
84	AR106	Hybrid	Open pollination	Public domain ^14^
85	GJ5	Hybrid	Open pollination	Public domain ^1^
86	GJ3	Hybrid	Open pollination	Public domain ^1^
87	BG180	Hybrid	Open pollination	Public domain ^12^
88	P50	Hybrid	Open pollination	Public domain ^5^
89	AS1	Hybrid	Open pollination	Public domain ^10^
90	AS5	Hybrid	Open pollination	Public domain ^10^
91	AS6	Hybrid	Open pollination	Public domain ^10^
92	AS7	Hybrid	Open pollination	Public domain ^10^
93	BAG41	Hybrid	Open pollination	AGB Embrapa
94	N1	Hybrid	Open pollination	Public domain ^3^
95	N16	Hybrid	Open pollination	Public domain ^3^
96	AS10	Hybrid	Open pollination	Public domain ^10^
97	AS2	Hybrid	Open pollination	Public domain ^10^
98	LB22	Hybrid	Open pollination	Public domain ^6^
99	LB88	Hybrid	Open pollination	Public domain ^6^
100	LB68	Hybrid	Open pollination	Public domain ^6^
101	LB33	Hybrid	Open pollination	Public domain ^6^
102	LB20	Hybrid	Open pollination	Public domain ^6^
103	LB102	Hybrid	Open pollination	Public domain ^6^
104	LB12	Hybrid	Open pollination	Public domain ^6^
105	LB110	Hybrid	Open pollination	Public domain ^6^
106	LB60	Hybrid	Open pollination	Public domain ^6^
107	LB15	Hybrid	Open pollination	Public domain ^6^
108	LB10	Hybrid	Open pollination	Public domain ^6^
109	GJ30	Hybrid	Open pollination	Public domain ^1^
110	B3T8P54	Hybrid	Emcapa03 × Robusta2258	AGB Embrapa
111	GJ8	Hybrid	Open pollination	Public domain ^1^
112	BAG28	Hybrid	Open pollination	AGB Embrapa
113	BAG19	Hybrid	Emcapa03 × Robusta1675	AGB Embrapa
114	BAG21	Hybrid	Robusta1675 × Cpafro194	AGB Embrapa
115	BRS3220	Hybrid	Emcapa03 × Robusta1675	Cultivar
116	GJ25	Hybrid	Open pollination	Public domain ^1^
117	BRS1216	Hybrid	Emcapa03 × Robusta1675	Cultivar
118	BAG26	Hybrid	Emcapa03 × Robusta2258	AGB Embrapa
119	BRS3213	Hybrid	Emcapa03 × Robusta2258	Cultivar
120	BAG24	Hybrid	Emcapa03 × Robusta1675	AGB Embrapa
121	BRS3210	Hybrid	Emcapa03 × Robusta2258	Cultivar
122	BAG33	Hybrid	Open pollination	AGB Embrapa
123	BAG34	Hybrid	Open pollination	AGB Embrapa
124	BAG23	Hybrid	Open pollination	AGB Embrapa
125	BAG22	Hybrid	Emcapa03 × Robusta2258	AGB Embrapa
126	VP148	Hybrid	Open pollination	Public domain ^5^
127	BRSOuroPreto120	Conilon	Open pollination	Cultivar
128	BRSOuroPreto125	Conilon	Open pollination	Cultivar
129	Con854	Conilon	Open pollination	AGB Embrapa
130	Con1048	Conilon	Open pollination	AGB Embrapa
131	BRS3137	Hybrid	Open pollination	Cultivar
132	BRS2336	Hybrid	Open pollination	Cultivar
133	BRSOuroPreto203	Conilon	Open pollination	Cultivar
134	BRS2299	Hybrid	Open pollination	Cultivar
135	BRSOuroPreto130	Conilon	Open pollination	Cultivar
136	BRSOuroPreto199	Hybrid	Open pollination	Cultivar
137	BRSOuroPreto184	Conilon	Open pollination	Cultivar
138	BRSOuroPreto155	Conilon	Open pollination	Cultivar
139	BRS3193	Hybrid	Open pollination	Cultivar
140	BRS2314	Hybrid	Emcapa03 × Robusta640	Cultivar

^1^ Geraldo Jacomin—Nova Brasilândia do Oeste, ^2^ Sergio Kalk—Cacoal, ^3^ Nivaldo Ferreira—Cacoal, ^4^ Carlos Alves da Silva—Novo Horizonte do Oeste, ^5^ Valdecir Piske—Alta Floresta do Oeste, ^6^ Laerte Braun—Nova Brasilândia do Oeste, ^7^ Alcides Rosa—Rolim de Moura, ^8^ Ronaldo Vitoriano—Alta Floresta do Oeste, ^9^ Ronaldo G Oliveira—Alta Floresta do Oeste, ^10^ Ademar Schmidt—Alta Floresta do Oeste, ^11^ Gilberto Boon—Alta Floresta do Oeste, ^12^ Adilson Berger—Rolim de Moura, ^13^ Wanderly Bernabé—Alto Alegre dos Parecis, ^14^ Aldinei Raasch—São Miguel do Guaporé, ^15^ Wanderley Peter—Cacoal.

**Table 3:** Molecular markers used in marker-assisted selection (MAS) and their association with genes conferring resistance to *Hemileia vastatrix* and *Colletotrichum kahawae*.

Resistance	Loci	Marker	Primers	Temp.	References
*Hemileia vastatrix*	A	SSR016	F: ACCCGAAAGAAAGAACCAAG R: CCACACAACTCTCCTCATTC	65	[22,23]
	B	CaRHv9	F: TGATGAAGAAGAGCGCATAGC R: GTCTAAGACCAGAATCAGATGG	65	[23]
	C	CARF005	F: GGACATCAACACCAACCTC R: ATCCCTACCATCCACTTCAAC	60	[8,15]
	D	RLK 2	F: GCTCACAGGTCCGATTCCTCTG R: TTTGGGAATAGGCCCGGAAAGA	66	[23]
*Colletotrichum kahawae*	E	Sat235	F: TCGTTCTGTCATTAAATCGTCAA R: GCAAATCATGAAAATAGTTGGTG	50	[24,25]
	F	Sat207	R: GAAGCCGTTTCAAGCC F: CAATCTCTTTCCGATGCTCT	50	[24,25]

F = Forward primer; R = Reverse; Temp. = annealing temperature.

## Data Availability

The original contributions presented in the study are included in the article.

## References

[1] Bilen C., El Chami D., Mereu V., Trabucco A., Marras S., Spano D. (2023). A Systematic Review on the Impacts of Climate Change on Coffee Agrosystems. Plants.

[2] Grüter R., Trachsel T., Laube P., Jaisli I. (2022). Expected global suitability of coffee, cashew and avocado due to climate change. PLoS ONE.

[3] Jawo T.O., Kyereh D., Lojka B. (2023). The Impact of Climate Change on Coffee Production of Small Farmers and Their Adaptation Strategies: A Review. Clim. Dev..

[4] Davis A.P. (2011). *Psilanthus mannii*, the Type Species of *Psilanthus*, Transferred to *Coffea*. Nord. J. Bot..

[5] Maurin O., Davis A.P., Chester M., Mvungi E.F., Jaufeerally-Fakim Y., Fay M.F. (2007). Towards a Phylogeny for *Coffea* (Rubiaceae): Identifying Well-Supported Lineages Based on Nuclear and Plastid DNA Sequences. Ann. Bot..

[6] Espindula M.C., Dalazen J.R., Rocha R.B., Teixeira A.L., Diocleciano J.M., Dias J.R.M., Schmidt R., Lima P.P., Lima G.M., Gama W. (2022). Robustas Amazônicos: Os Cafeeiros Cultivados em Rondônia.

[7] Silva A.N.R., Rocha R.B., Teixeira A.L., Espindula M.C., Partelli F.L., Caixeta E.T. (2024). Self-Incompatibility and Pollination Efficiency in *Coffea canephora* Using Fluorescence Microscopy. Agronomy.

[8] Alkimim E.R., Caixeta E.T., Sousa T.V., Silva F.L., Sakiyama N.S., Zambolim E.M., Pereira A.A., Oliveira A.C.B., de Souza F.F. (2020). Selective Efficiency of Genome-Wide Selection in *Coffea canephora* Breeding. Tree Genet. Genomes.

[9] Ferrão L.F.V., Caixeta E.T., Souza F.D.F., Zambolim E.M., Sakiyama N.S., Zambolim L., Cruz C.D., Pereira A.A. (2013). Comparative Study of Different Molecular Markers for Classifying and Establishing Genetic Relationships in *Coffea canephora*. Plant Syst. Evol..

[10] Rocha R.B., Teixeira A.L., Ramalho A.R., Espindula M.C., Lunz A.M.P., Souza F.F. (2021). *Coffea canephora* Breeding: Estimated and Achieved Gains from Selection in the Western Amazon, Brazil. Ciênc. Rural.

[11] Silva L.d.F., Leichtweis B.G., Silva A.C.A., Rocha R.B., Teixeira A.L., Caixeta E.T. (2024). Fingerprinting Amazonian coffees: Assessing diversity through molecular markers. Euphytica.

[12] Faria L.S., Alkimim E.R., Barreiro P.R.R.M., Caixeta E.T., Zambolim E.M., Oliveira A.C.B., Pereira A.A., Sakiyama N.S., de Souza F.F. (2022). Genome-Wide Association Study of Plant Architecture and Disease Resistance in *Coffea canephora*. Euphytica.

[13] Oliveira L.N.L., Rocha R.B., Ferreira F.M., Spinelli V.M., Ramalho A.R., Teixeira A.L. (2018). Selection of *Coffea canephora* Parents from the Botanical Varieties Conilon and Robusta for the Production of Intervarietal Hybrids. Ciênc. Rural.

[14] Teixeira A.L., Souza F.D.F., Rocha R.B., Junior J.R.V., Torres J.D., Rodrigues K.M., Moraes M.S., Silva C.A., Oliveira V.E.G., Lourenço J.L.R. (2017). Performance of Intraspecific Hybrids (Kouillou × Robusta) of *Coffea canephora* Pierre. Afr. J. Agric. Res..

[15] Alkimim E.R., Caixeta E.T., Sousa T.V., Silva F.L., Zambolim E.M., Pereira A.A., Oliveira A.C.B., Sakiyama N.S., de Souza F.F. (2021). Designing the Best Breeding Strategy for *Coffea canephora*: Genetic Evaluation of Pure and Hybrid Individuals Aiming to Select for Productivity and Disease Resistance Traits. PLoS ONE.

[16] Teixeira A.L., Rocha R.B., Espindula M.C., Ramalho A.R., Vieira Júnior J.R., Alves E.A., Lunz A.M.P., Souza F.F., Costa J.N.M., Fernandes C.d.F. (2020). Amazonian Robustas: New *Coffea canephora* Coffee Cultivars for the Western Brazilian Amazon. Crop Breed. Appl. Biotechnol..

[17] Leroy T., Marraccini P., Dufour M., Montagnon C., Lashermes P., Sabau X., Ferreira L.P., Jourdan I., Pot D., Andrade A.C. (2005). Construction and Characterization of a *Coffea canephora* BAC Library to Study the Organization of Sucrose Biosynthesis Genes. Theor. Appl. Genet..

[18] Ramalho A.R., Rocha R.B., Souza F.F., Veneziano W., Teixeira A.L. (2016). Genetic Progress of Processed Coffee Yield with the Selection of Conilon Coffee Clones. Rev. Ciênc. Agron..

[19] Viencz T., Acre L.B., Rocha R.B., Alves E.A., Ramalho A.R., Benassi M.T. (2023). Caffeine, Trigonelline, Chlorogenic Acids, Melanoidins, and Diterpenes Contents of *Coffea canephora* coffees Produced in the Amazon. J. Food Compos. Anal..

[20] Velásquez S., Banchón C. (2022). Influence of Pre- and Post-Harvest Factors on the Organoleptic and Physicochemical Quality of Coffee: A Short Review. J. Food Sci. Technol..

[21] Companhia Nacional de Abastecimento (Conab) (2024). Acompanhamento da Safra Brasileira de Café—Terceiro Levantamento—Setembro 2024.

[22] Alkimim E.R., Caixeta E.T., Sousa T.V., da Silva F.L., Sakiyama N.S., Zambolim L. (2018). High-throughput targeted genotyping using next-generation sequencing applied in *Coffea canephora* breeding. Euphytica.

[23] Alkimim E.R., Caixeta E.T., Sousa T.V., Pereira A.A., de Oliveira A.C.B., Zambolim L., Sakiyama N.S. (2017). Marker-assisted selection provides arabica coffee with genes from other *Coffea* species targeting on multiple resistance to rust and coffee berry disease. Mol. Breed..

[24] Almeida D.P., Caixeta E.T., Moreira K.F., Oliveira A.C.B., Zambolim E.M., Sakiyama N.S., Pereira A.A. (2021). Marker-Assisted Pyramiding of Multiple Disease Resistance Genes in Coffee Genotypes (*Coffea arabica*). Agronomy.

[25] Alvarenga S.M., Caixeta E.T., Hufnagel B., Maciel-Zambolim E., Zambolim L., Pereira A.A., Sakiyama N.S. (2011). Marcadores Moleculares Derivados de Sequências Expressas do Genoma Café Potencialmente Envolvidas na Resistência à Ferrugem. Pesqui. Agropecu. Bras..

[26] Combes M.C., Andrzejewski S., Anthony F., Bertrand B., Rovelli P., Lashermes P. (2000). Characterization of Microsatellite Loci in *Coffea arabica* and Related Coffee Species. Mol. Ecol..

[27] Caixeta E.T., Oliveira A.C.B., Brito G.G., Sakiyama N.S., Zambolim E.M., Pereira A.A., Borem A.L., Caixeta E.T. (2016). Tipos de Marcadores Moleculares. Marcadores Moleculares.

[28] Barka G.D., Caixeta E.T., Ferreira S.S., Zambolim E.M., Pereira A.A., Oliveira A.C.B., Sakiyama N.S. (2020). In Silico Guided Structural and Functional Analysis of Genes with Potential Involvement in Resistance to Coffee Leaf Rust: A Functional Marker Based Approach. PLoS ONE.

[29] Carneiro M.S., Vieira M.L.C. (2002). Genetic Maps in Plants. Bragantia.

[30] Ferrão M.A.G., da Fonseca A.F., Volpi P.S., de Souza L.C., Comério M., Filho A.C.V., Riva-Souza E.M., Munoz P.R., Ferrão R.G., Ferrão L.F.V. (2023). Genomic-Assisted Breeding for Climate-Smart Coffee. Plant Genome.

[31] Gichuru E.K., Agwanda C.O., Combes M.C., Mutitu E.W., Ngugi E.C.K., Bertrand B., Lashermes P. (2008). Identification of Molecular Markers Linked to a Gene Conferring Resistance to Coffee Berry Disease (*Colletotrichum kahawae*) in *Coffea arabica*. Plant Pathol..

[32] Gichuru E., Alwora G., Gimase J., Kathurima C. (2021). Coffee Leaf Rust (*Hemileia vastatrix*) in Kenya—A Review. Agronomy.

[33] Zambolim L., Caixeta E.T. (2021). An Overview of Physiological Specialization of Coffee Leaf Rust—New Designation of Pathotypes. Int. J. Curr. Res..

[34] Capucho A.S., Zambolim E.M., Freitas R.L., Oliveira A.C.B., Sakiyama N.S., Pereira A.A. (2012). Identification of Race XXXIII of *Hemileia vastatrix* on *Coffea arabica* Catimor Derivatives in Brazil. Australas. Plant Dis. Notes.

[35] Diniz L.E., Sakiyama N.S., Lashermes P., Caixeta E.T., Oliveira A.C.B., Zambolim E.M., Pereira A.A. (2005). Analysis of AFLP Markers Associated to the *Mex-1* Resistance Locus in *Icatu* Progenies. Crop Breed. Appl. Biotechnol..

[36] Pestana K.N., Capucho A.S., Caixeta E.T., Almeida D.P., Zambolim E.M., Cruz C.D., Zambolim L., Pereira A.A., Oliveira A.C.B., Sakiyama N.S. (2015). Inheritance Study and Linkage Mapping of Resistance Loci to *Hemileia vastatrix* in Híbrido de Timor UFV 443-03. Tree Genet. Genomes.

[37] Zullo J., Pinto H.S., Assad E.D., Ávila A.M.H. (2011). Potential for growing Arabica coffee in the extreme south of Brazil in a warmer world. Clim. Change.

[38] Hoque A., Fiedler J.D., Rahman M. (2020). Genetic Diversity Analysis of a Flax (*Linum usitatissimum* L.) Global Collection. BMC Genom..

[39] Sousa T.V., Caixeta E.T., Alkimim E.R., Oliveira A.C.B., Pereira A.A., Sakiyama N.S., Resende M.D.V., Zambolim L. (2017). Population Structure and Genetic Diversity of Coffee Progenies Derived from Catuaí and Híbrido de Timor Revealed by Genome-Wide SNP Marker. Tree Genet. Genomes.

[40] Talhinhas P., Batista D., Diniz I., Vieira A., Silva D.N., Loureiro A., Tavares S., Pereira A.P., Azinheira H.G., Guerra-Guimarães L. (2017). The Coffee Leaf Rust Pathogen *Hemileia vastatrix*: One and a Half Centuries Around the Tropics. Mol. Plant Pathol..

[41] Vieira A., Diniz I., Loureiro A., Pereira A.P., Silva M.C., Várzea V., Batista D. (2019). Aggressiveness Profiling of the Coffee Pathogen *Colletotrichum kahawae*. Plant Pathol..

[42] Mahé L., Combes M.C., Várzea V.M.P., Guilhaumon C., Lashermes P. (2008). Development of Sequence-Characterized DNA Markers Linked to Leaf Rust (*Hemileia vastatrix*) Resistance in Coffee (*Coffea arabica* L.). Mol. Breed..

[43] Saavedra L.M., Caixeta E.T., Barka G.D., Borém A., Zambolim L., Nascimento M., Cruz C.D., Oliveira A.C.B.d., Pereira A.A. (2023). Marker-Assisted Recurrent Selection for Pyramiding Leaf Rust and Coffee Berry Disease Resistance Alleles in *Coffea arabica* L. Genes.

[44] Silva M.d.C., Guerra-Guimarães L., Diniz I., Loureiro A., Azinheira H., Pereira A.P., Tavares S., Batista D., Várzea V. (2022). An Overview of the Mechanisms Involved in Coffee-*Hemileia vastatrix* Interactions: Plant and Pathogen Perspectives. Agronomy.

[45] Denoeud F., Carretero-Paulet L., Dereeper A., Droc G., Guyot R., Pietrella M., Zheng C., Alberti A., Anthony F., Aprea G. (2014). The coffee genome provides insight into the convergent evolution of caffeine biosynthesis. Nat. Genet..

[46] Zaidan I.R., Ferreira A., Noia L.R., Santos J.G., Arruda V.C., Couto D.P.D., Braz R.A., Senra J.F.B., Partelli F.L., Azevedo C.F. (2023). Diversity and structure of *Coffea canephora* from old seminal crops in Espírito Santo, Brazil: Genetic resources for coffee breeding. Tree Genet. Genomes.

[47] Gnirke A., Melnikov A., Maguire J., Rogov P., LeProust E.M., Brockman W., Fennell T., Giannoukos G., Fisher S., Russ C. (2009). Solution Hybrid Selection with Ultra-Long Oligonucleotides for Massively Parallel Targeted Sequencing. Nat. Biotechnol..

[48] Nei M., Li W.H. (1979). Mathematical Model for Studying Genetic Variation in Terms of Restriction Endonucleases. Proc. Natl. Acad. Sci. USA.

[49] Rocha R.B., Suela M.M., Comério M., Souza E.M.R., Senra J.F.B., Ferrão M.A.G., Ferrão R.G., Fonseca A.F.A., Filho A.C.V., Volpi P.S. (2025). Genomic-assisted selection to guide mate allocation and leverage hybrid vigor in *Coffea canephora*. Tree Genet. Genomes.

[50] Jombart T., Devillard S., Balloux F. (2010). Discriminant Analysis of Principal Components: A New Method for the Analysis of Genetically Structured Populations. BMC Genet..

